# Le lymphangiome kystique rétropéritonéal: à propos de 5 cas et revue de la littérature

**DOI:** 10.11604/pamj.2016.25.73.10002

**Published:** 2016-10-06

**Authors:** Ahmed Saadi, Haroun Ayed, Omar Karray, Walid Kerkeni, Abderrazak Bouzouita, Mohamed Cherif, Riadh Ben Slama, Amine Derouiche, Mohamed Chebil

**Affiliations:** 1 Service d’Urologie, Hôpital Charles-Nicolle, Faculté de Médecine de Tunis, Université de Tunis El Manar, boulevard 9-Avril, 1006 Tunis, Tunisie

**Keywords:** Lymphangiome kystique rétropéritonéal, diagnostic, traitement, chirurgie, Retroperitoneal cystic lymphangioma, diagnosis, treatment, surgery

## Abstract

Le lymphangiome kystique est une tumeur bénigne malformative rare des vaisseaux lymphatiques à localisations diverses. La localisation rétropéritonéale est moins fréquente comparée à celle mésentérique. Sa présentation clinique est polymorphe. Le diagnostic est évoqué par l'imagerie mais il nécessite une confirmation histologique. Le traitement de choix est chirurgical. Notre objectif est d'étudier les manifestations cliniques, les complications, les aspects diagnostiques et thérapeutiques de cette tumeur. Nous rapportons une série de 5 cas de lymphangiomes kystiques rétropéritonéaux (4 femmes et un homme) opérés dans notre service entre les années 2004 et 2014. Leurs dossiers ont été examinés rétrospectivement. Le suivi était basé sur l'examen clinique et l'échographie abdominale. L´âge moyen était de 45 ans. Le suivi moyen était de 32,6 mois. La symptomatologie révélatrice la plus fréquente était les douleurs et/ou une masse abdominale. Le scanner abdominal était l'examen le plus utile au diagnostic. Une exérèse complète était réalisée d'emblée chez quatre patients et elle était différée après cinq ans de surveillance par une échographie annuelle chez un. Dans un cas, on a eu recours à une néphrectomie. Aucune récidive ni complication n´ont été notées chez les 5 patients. le lymphangiome kystique à localisation rétropéritonéale est une affection rare. Sa prise en charge thérapeutique repose sur une exérèse complète, de cas de lésions symptomatiques ou de complications, pour limiter le risque de récidive. Cette dernière peut être différée chez les patients asymptomatiques.

## Abstract (en)

Cystic lymphangioma is a rare, benign malformation of the lymphatic vessels which may be observed on various locations. Retroperitoneal location is less common than mesenteric location. Cystic lymphangioma has a polymorphic clinical presentation. Diagnosis is based on imaging but requires histological confirmation. Surgery is the treatment of choice. The aim of our study is to analyze the clinical manifestations, complications, diagnostic and therapeutic aspects of this tumor. We report a case series of 5 patients with retroperitoneal cystic lymphangioma (4 women and 1 man) operated in our department between the years 2004 and 2014. Their medical records were reviewed retrospectively. Follow-up was based on clinical examination and abdominal CT scan. The average age was 45 years. The mean follow-up was 32.6 months. The most common symptoms indicative of retroperitoneal cystic lymphangioma were pains and/or an abdominal mass. Abdominal CT scan was the most useful diagnostic test. Total resection was immediately achieved in 4 patients and it was deferred for up to 5 years in one patient. He underwent annual ultrasound monitoring. One patient underwent nephrectomy. No recurrence or complications were noted in 5 patients. Retroperitoneal cystic lymphangioma is a rare condition. Its therapeutic management is based on complete resection in patients with symptomatic lesions or complications, in order to limit the risk of recurrence. Complete resection may be deferred in asymptomatic patients.

## Introduction

Le lymphangiome kystique est une tumeur malformative bénigne du système lymphatique [1–4] touchant le plus souvent la région crânio-faciale, cervicale ou thoracique, et découverte habituellement dans l'enfance [3, 4]. Sa localisation abdominale et surtout rétro péritonéale est plus rare [3, 4]. La présentation clinique est très polymorphe [3, 4]. Le diagnostic est évoqué par l'imagerie mais il nécessite une confirmation histologique [1, 3]. Son traitement est chirurgical et n´est pas toujours simple [3].

## Méthodes

Nous avons mené une étude rétrospective de cinq cas de lymphangiomes kystiques rétro péritonéaux opérés dans notre service entre les années 2004 et 2014. Nous avons recueilli, pour chaque malade, les particularités épidémiologiques, cliniques, radiologiques, thérapeutiques et évolutives.

## Résultats

**Figure 1:**
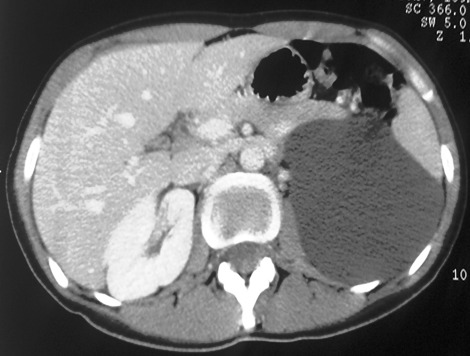
Coupe axiale tomodensitométrique après injection de produit de contraste en intraveineux, montrant le lymphangiome kystique qui est hypodense, homgène et ne prenant pas le contraste

**Figure 2:**
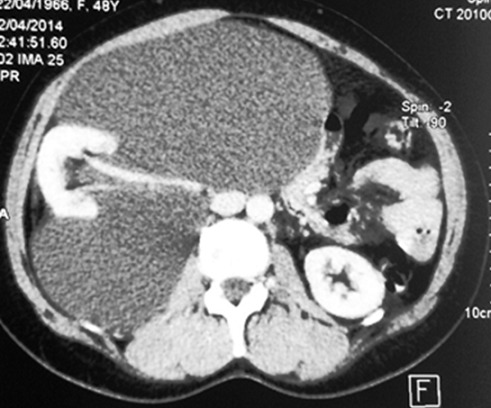
Coupe axiale tomodensitométrique: le lymphangiome kystique qui refoule le rein droit en haut et en avant et s’insinue autours du pédicule rénal

**Figure 3:**
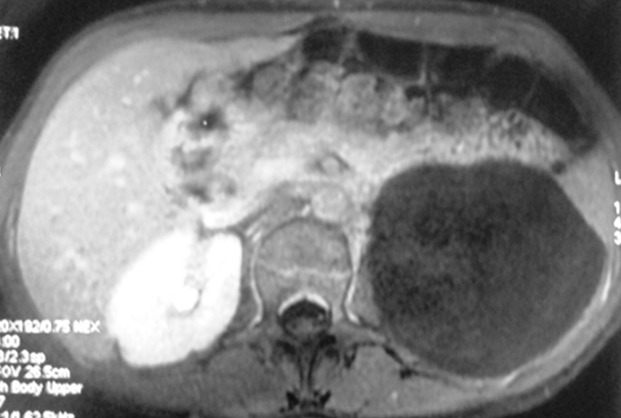
Coupe axiale d’une IRM: volumineuse masse kystique rétropéritonéale gauche en hyposignal T1 assez homogène

**Figure 4:**
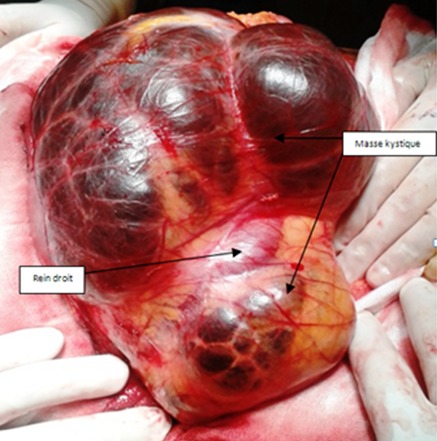
Aspect per-opératoire: volumineuse masse kystique polylobée s’insinuant autours du rein et du pédicule rénal droit, opérée par voie sous costale droite

Il s'agit de 4 femmes et un homme. Leur âge moyen était de 45 ans (30-74 ans). Quatre patientes se sont présentées pour douleurs et/ou pesanteur avec une masse abdominale ou lombaire associée. Un patient était asymptomatique avec découverte échographique fortuite d'une masse rétro péritonéale. L'examen physique était normal chez tous les patients, hormis une masse abdominale palpable. Tous les patients ont été explorées par une échographie abdomino-pelvienne et un examen tomodensitométrique. Une IRM a été pratiquée chez une seule patiente. La tumeur était toujours unilatérale, droite dans 3 cas, gauche dans un cas et pelvienne dans un cas. L'échographie a montré une tumeur liquidienne bien limitée uniloculaire dans 3 cas et multiloculaire chez les 2 autres. Sur le scanner, la tumeur était spontanément hypodense, bien limitée et à contenu homogène, ne prenant pas le contraste, tout comme ses cloisons qui sont fines (Figure 1). Dans un cas, la masse kystique refoulait le rein droit en haut et en avant et s'insinue autours du pédicule rénal (Figure 2). Le diamètre moyen de la tumeur était de 14 cm (10-20 cm). Une imagerie par résonance magnétique complémentaire a été réalisée dans un cas, ayant objectivé une masse kystique de signal liquidien avec un hyposignal en T1 et un hypersignal enT2 homogène, et l'absence de prise de contraste après injection de gadolinium (Figure 3). Le diagnostic de certitude n'a pu être établi qu'après examen histologique de la pièce opératoire. Une kystectomie à ciel ouvert a été réalisée dans tous les cas: par lombotomie dans deux cas, par une incision médiane dans deux cas et par une incision sous costale droite dans un cas. L'exploration a trouvé une masse kystique à paroi fine, à contenu liquidien jaune citrin, uni ou polylobée, qui refoule les organes de voisinages. Dans un cas, la masse s'insinue autours du pédicule rénal et du rein doit et était accolée à la veine cave inférieure (Figure 4). Une exérèse complète de la masse kystique a été réalisée dans tous les cas. Une néphrectomie a été associée dans un cas. Les suites opératoires étaient simples dans tous les cas. La durée moyenne du séjour post-opératoire était de 4,2 jours (3-6 jours). Dans un cas, la chirurgie a été différée devant le caractère pauci-symptomatique du kyste et l'absence de signes de complications, le patient a été surveillé par des échographies annuelles pendant 5ans jusqu´à ce qu´il présente une aggravation de la symptomatologie (douleurs pelviennes avec dysurie et pollakiurie) avec augmentation du volume du kyste ( de 10 à 16cm) mais qui a gardé les mêmes caractéristiques scannographiques sans signes de complications. Le suivi était basé sur l'examen clinique et l'échographie abdominale. Aucune récidive n'a été observée avec un recul moyen de 32,6 mois (7-60 mois).

## Discussion

Les lymphangiomes kystiques sont des tumeurs bénignes rares [1, 2] qui se voient surtout chez l´enfant [3]. La moitié de ces lésions serait présente à la naissance, et 90 % des lymphangiomes kystiques se développeraient jusqu'à l'âge de 2 ans [4]. Le développement chez l´adulte est exceptionnel [3, 5]. Les hommes et les femmes seraient atteints de manière semblable à l'âge adulte [4], alors que chez l'enfant, le sexe ratio est, soit semblable [4], soit légèrement prédominant chez le garçon [4]. Dans notre série on a noté une prédominance féminine. L'hypothèse la plus probable du développement d'un lymphangiome kystique serait une origine malformative congénitale [4]. Il est admis que le système lymphatique périphérique se développe à partir de sacs primitifs issus du système veineux. La formation d'un kyste est l'évolution d'un bourgeon lymphatique secondaire à un défaut de connexion, lors de l'embryogenèse, entre des chaînes lymphatiques et le système veineux [3, 4]. Les lymphangiomes kystiques sont considérés donc des tumeurs vasculaires malformatives pour lesquelles il n'a encore jamais été démontré de potentiel de malignité [4]. L'origine acquise a été évoquée résultant d´une obstruction des vaisseaux lymphatiques à la suite d´une inflammation, d´un traumatisme ou d´une dégénérescence [3, 6]. Les zones les plus fréquemment touchées sont les tissus sous-cutanés du cou (environ 75 %) et des aisselles (environ 15 %) [4]. Les localisations médiastinales et abdominales sont beaucoup plus rares, environ 10 % des cas [4]. Le lymphangiome kystique représente 7 % des lésions kystiques abdominales chez l'adulte [3, 4]. Les lésions touchent alors essentiellement le mésentère, mais peuvent concerner également le tractus gastro-intestinal, la rate, le foie, les reins, les surrénales et le pancréas [4]. La localisation rétropéritonéale est moins fréquente que la localisation mésentérique [1–5]. Les manifestations cliniques du lymphangiome kystique abdominal sont très polymorphes [3, 4]. La majorité des lymphangiomes kystiques abdominaux (jusqu'à 60 % 4,7) sont retrouvés avant l'âge de 5 ans. Des lésions se développant plus lentement, notamment rétropéritonéales, peuvent ne se manifester qu'à l'âge adulte sous forme d´une masse souvent asymptomatique [3, 4]. Un volume tumoral important provoque habituellement des douleurs abdominales, symptôme le plus fréquent, mais peut aussi entraîner une augmentation du périmètre abdominal, une masse palpable, une occlusion intestinale voire un volvulus [4]. Le symptôme le plus courant dans notre série était l'apparition de douleurs avec masse abdominales. D'autres complications peuvent également provoquer des tableaux cliniques aigus : une hémorragie intra-kystique, une surinfection ou une hémorragie digestive, cette dernière étant toutefois peu commune [4]. La rupture spontanée des kystes est rare [4]. Enfin, il faut mentionner une forme rarissime de la maladie : la lymphangiomatose kystique péritonéale, qui réalise une atteinte disséminée pouvant mimer une carcinose péritonéale, et associée à des lésions médiastinales et à un chylothorax [4].

Devant l'absence de signes cliniques spécifiques, le bilan radiologique va orienter le diagnostic [7]. Pour établir le diagnostic, l'échographie est l'examen le plus utile initialement [4]. Elle montre classiquement une tumeur liquidienne uni ou multiloculaire avec des cloisons fines qui est bien limitée [3]. Le contenu des kystes, souvent transonore, peut cependant devenir échogène à l´occasion d´une hémorragie intra-kystique voire contenir quelques calcifications [3, 8]. Dans nos observations, l'échographie a permis de détecter la lésion dans 100% des cas et les kystes étaient uniloculaires chez 3 de nos patients et cloisonné chez les 2 autres. Aucun signe de complications n'a été retrouvé. Ces aspects échographiques restent non spécifiques et le scanner présente un excellent moyen diagnostique initial chez l'adulte [3, 4]. Il montre habituellement une tumeur à contenu homogène, hypodense, ne prenant pas le contraste, tout comme ses cloisons qui sont fines et il permet d´étudier la densité de la tumeur. Le scanner permet aussi d´évaluer les rapports de la tumeur avec les organes de voisinage et de différencier le lymphangiome rétro-péritonéal du lymphangiome intra-péritonéal [1]. L'IRM, en seconde intention, permet de mieux préciser la nature du contenu des kystes [4] et apprécie très bien l´extension périvasculaire de la lésion [3]. Le lymphangiome kystique est de signal liquidien : en hyposignal en T1 et hypersignal en T2. Les cloisons sont en hyposignal en T1 et T2. L'injection de gadolinium ne montre que peu ou pas de rehaussement pariétal et septal [9]. D'autres investigations radiologiques ont été rapportées dans la littérature, dont la lympho-scintigraphie à l'albumine qui ne semble pas apporter d'élément diagnostique supplémentaire, le lymphangiome ne communiquant pas ou peu avec le système lymphatique périphérique [4, 8]. Si les lésions de la sphère cervico-faciale chez l'enfant permettent souvent de poser le diagnostic simplement sur la clinique et la radiologie, les atteintes intra-abdominales peuvent parfois poser plus de difficultés [4]. Le diagnostic différentiel comprend le lymphome, une duplication digestive ou un volumineux kyste ovarien [4]. Des lymphocèles post- opératoires peuvent également avoir un aspect radiologique similaire, mais le siège et le contexte étiologique particulier évoquent le diagnostic [4]. Aucun de nos cas n'a un antécédent de chirurgie susceptible de se compliquer de lymphocèle post- opératoire.

La preuve définitive du diagnostic de lymphangiome kystique est apportée par l'examen anatomopathologique [3, 4]. On distingue habituellement les formes oligo-macrokystiques, micro-poly-kystiques et mixtes [4]. Au microscope, les critères diagnostiques sont: des vaisseaux lymphatiques dilatés, bordés de cellules endothéliales aplaties sans signes d'atypies, avec présence d'un tissu lymphoïde abondant [4]. Des cellules musculaires lisses et des cellules spumeuses contenant du matériel lipidique peuvent être observées au niveau de la paroi de ces vaisseaux [4]. Le diagnostic histologique est parfois impossible sur de simples biopsies en présence de remaniements avec disparition de l´endothélium et apparition de dépôts de fibrine secondaire à l'inflammation et l'hémorragie [3]. Les lésions n'entraînant pas de gêne pour le patient doivent être suivies par des imageries répétées [4]. En effet, certaines lésions peuvent régresser spontanément, et les différents traitements disponibles peuvent tous entraîner des complications de gravité variable [4]. Un de nos patient a été initialement surveillé pendant 5ans par des échographies annuelles devant le caractère pauci-symptomatique du kyste initialement et l'absence de signes de complications et il n'a été traité que devant l'aggravation des symptômes suite à l'augmentation du volume du kyste. L'aspiration du contenu du kyste, avec ou sans injection de produit sclérosant, a des résultats à long terme variés : les récidives semblent fréquentes, jusqu'à 100 % dans certaines séries [4, 10]. L´exérèse chirurgicale, ouverte ou laparoscopique, est l´attitude classique dans la localisation abdominale [4, 11, 12]. L'exérèse doit être la plus complète possible pour éviter les récidives [3], en restant conservateur pour les autres organes, vu le caractère bénin du lymphangiome [4]. Dans un cas on eu recours à une néphrectomie associée à l'exérèse du kyste vu les accolements au rein, au pédicule rénal et la veine cave inférieure. Afin de limiter les complications par fuite lymphatique postopératoire (lymphocèle, ascite chyleuse), la lymphostase doit être minutieuse, soit par ligatures pas à pas, soit peut-être avec l'UltraCision ou le LigaSure^™^[4]. Le taux de récidive est de 40 % après résection incomplète et de 17 % après résection macroscopiquement complète, toutes localisations confondues [4, 10]. Les lésions lymphangiomateuses seraient ainsi beaucoup plus étendues qu'on ne pourrait le penser [4]. Aucune récidive n'a été détectée chez nos 5 patients avec un recul moyen de 32,6 mois.

## Conclusion

Le lymphangiome kystique est une tumeur bénigne du système lymphatique qui se voie surtout chez l´enfant. La localisation rétropéritonéale est rare. La présentation clinique est très polymorphe. Le diagnostic est suspecté par l'imagerie et ne peut être confirmé que lors de l´examen histologique après l'intervention. En cas de lésion non symptomatique, la surveillance est la règle. En cas de lésion symptomatique, l'exérèse chirurgicale complète semble la meilleure option pour limiter le risque de récidive.

### Etat des connaissances actuelles sur le sujet

- Les lymphangiomes kystiques sont des tumeurs rares qui se voient surtout chez l'enfant.
- Le diagnostic est évoqué sur la radiologie et confirmé par l'histologie.
- Le traitement est chirurgical.

### Contribution de notre étude à la connaissance

- Description des particularités épidémiologiques, cliniques, radiologiques, thérapeutiques et évolutives de cette pathologie chez l'adulte à travers notre série et une revue de la littérature.
- Discussion des indications thérapeutiques.
